# The Relationship between Neighbourhood Green Space and Child Mental Wellbeing Depends upon Whom You Ask: Multilevel Evidence from 3083 Children Aged 12–13 Years

**DOI:** 10.3390/ijerph14030235

**Published:** 2017-02-27

**Authors:** Xiaoqi Feng, Thomas Astell-Burt

**Affiliations:** 1 Population Wellbeing and Environment Research Lab (PowerLab), School of Health and Society, University of Wollongong, Wollongong, NSW 2522, Australia; thomasab@uow.edu.au; 2 Early Start Research Institute, University of Wollongong, Wollongong, NSW 2522, Australia; 3 Illawarra Health and Medical Research Institute, University of Wollongong, Wollongong, NSW 2522, Australia

**Keywords:** child health, mental wellbeing, green space quantity, green space quality, Strengths and Difficulties Questionnaire, Australia

## Abstract

Recent reviews of the rapidly growing scientific literature on neighbourhood green space and health show strong evidence for protective and restorative effects on mental wellbeing. However, multiple informants are common when reporting mental wellbeing in studies of children. Do different informants lead to different results? This study utilised nationally representative data on Goodman’s 25-item Strengths and Difficulties Questionnaire reported by 3083 children (aged 12–13 years old), and their parents and teachers. Multilevel models were used to investigate whether similar associations between child mental wellbeing (as measured using the total difficulties score and the internalising and externalising subscales) and neighbourhood green space quantity and quality are obtained regardless of the informant. After adjustment for confounders, higher green space quantity and quality were associated with consistently more favourable child mental wellbeing on all three measures, regardless of the informant. However, associations with green space quantity were statistically significant (*p* < 0.05) only for the parent-reported total difficulties score and the internalising subscale. Significant associations with green space quality were consistently observed for both parent- and child-reported outcomes. Teacher-reported outcomes were not significantly associated with green space exposure. Future studies of green space and child health should acknowledge when different informants of outcomes could lead to different conclusions.

## 1. Introduction

Recent reviews of the rapidly growing scientific literature on neighbourhood green space and health show strong evidence for protective and restorative effects on mental wellbeing [1,2,3]. Findings have been reasonably consistent irrespective of the measure of green space exposure, though fairly recent evidence suggests that different results may be contingent upon the way in which the health construct is measured. Take, for example, the relationship between green space and mental wellbeing among adults. A fairly recent cross-sectional study examined the benefits of participating in physical activity within green spaces compared to physical activity in non-green locations [4]. The main outcome was mental wellbeing measured using the General Health Questionnaire (GHQ) [5] and the Warwick-Edinburgh Mental Well-Being Scale (WEMWBS) [6]. Expected results were found for the former, but not the latter. This is likely to be due to the GHQ and WEMWBS measuring different aspects of mental wellbeing. This example clearly underlines the importance of theoretically driven selections of outcome indicators to measure the health construct and, where relevant and if data permits, to analyse multiple (or replicate with alternative) outcome variables in order to triangulate, validate and bolster the case for causal inferences and social policy impacts. 

Much the same could be said for studies of the relationship between neighbourhood green space and child health and developmental trajectories. These are fewer in number compared to studies of adult health, but are nonetheless an important focus of current research since children are more sensitive to impacts of environmental exposures due to this being a period of rapid growth and development [7]. It is also important from the perspective of lifecourse epidemiology, through which it is now widely recognised that early life experiences and exposures can have important developmental effects on life-long health trajectories [8]. 

Extending the earlier example of adult mental wellbeing to children is an especially interesting case requiring replication of findings since, unlike for adults, there are many examples where the information used to derive those indicators for children is reported by different informants. Some studies use outcome data reported by the child, such as Huynh and colleagues’ analysis of Canadian children aged 11 to 16 years old in which positive emotional wellbeing was associated with green space availability [9]. However, it seems to be more common among the green space and child health literature to utilise indicators of mental wellbeing for which the underlying information is reported by a parent. For example, Goodman’s 25-item Strengths and Difficulties Questionnaire (SDQ) is an internationally recognised behavioural screening tool of child wellbeing [10]. A cursory review of the literature shows the parent-reported version of the SDQ has been used to examine associations between mental wellbeing and neighbourhood green space among children in Germany [11], Lithuania [12], Spain [13] and England [14]. All have reported protective associations.

Although prior studies of neighbourhood green space and SDQ-derived outcomes have tended to rely on parent-report, the SDQ can be filled in by the children themselves and also by their teachers [15]. This raises the intriguing question of whether broadly similar results are obtained in analyses of green space and child mental wellbeing when using the same outcome for the same sample, but reported by different informants? Answering this question is the purpose of this study, which will then provide insights into the reliability of the previously observed association when all characteristics of the study sample remain identical but that which informs the outcome variable is permitted to vary.

## 2. Methods

### 2.1. Data

This study makes use of the wave 5 data collection for the “K cohort” of the Longitudinal Study of Australian Children (LSAC) that took place in 2012 [16]. This was the first wave of the LSAC in which the Goodman’s 25-item Strengths and Difficulties Questionnaire (SDQ) was filled in by parents, children and teachers. Previously parents and teachers were the sole informants. A total of 3956 children participated in wave 5, which is approximately 79.4% of the original sample recruited at wave 1 in 2004 (*n* = 4983). Recruitment at wave 1 was from the Medicare enrolment database, which is the most comprehensive data of Australia’s population and supported attempts to create a nationally representative sample. One child per family was permitted to take part. A spatially clustered design was used based upon postcodes across states and territories in order to make face-to-face interviews with families cost-effective [17]. Participants’ home addresses were geocoded and linked by the data custodian to the “Statistical Area 2” (SA2) area identifier. The SA2 was developed by the Australian Bureau of Statistics to be a geographical approximation of local community. SA2s contain about 10,000 residents on average [18]. Of the 3956 participants in wave 5, 872 were omitted due to non-response on at least one of the outcome variables. One additional participant was omitted due to a missing SA2 identifier, making the final analytical sample 3083 participants nested within 1106 SA2s.

### 2.2. Child Mental Wellbeing

Goodman’s 25-item Strengths and Difficulties Questionnaire (SDQ) is a behavioural screening tool of child wellbeing that is commonly filled in by parents or children, but can also be filled in by teachers [10]. In this study analyses are conducted on SDQ data reported by the lead spokes-parent (usually the biological mother), the child themselves and their teacher. The SDQ comprises five scales: emotional symptoms; conduct problems; hyperactivity; peer problems; and prosocial behavior [19]. It is validated for use in multi-cultural settings [20]. Summing the emotional symptoms, conduct problems, hyperactivity and peer problems scales creates a composite variable known as the total difficulties score (TDS). The TDS is the primary outcome variable in this study. It ranges from 0 to 40, with higher scores representing increasing difficulties and an increased probability of clinician-assigned mental disorder [10].

In line with previous research involving the SDQ, two subscales were also analysed as secondary outcome variables. The first subscale was the “internalizing subscale”, which was derived by summing the emotional and peer symptoms scales. The second was the “externalizing subscale”, which was derived by summing the conduct problems and hyperactivity scales. Separate analyses of internalising and externalising subscales was considered to add potential value through insights into the extent that neighbourhood green space is associated with negative emotional states that might be internalised (e.g., nervousness, worry, anxiety, depression) or problems that are externalised (e.g., fidgetiness, impulsiveness and a lack of concentration). Previous work has already demonstrated the construct validity of the internalising and externalising subscales [15]. 

### 2.3. Green Space Exposures

Epidemiological studies on green space and health typically examine the quantity of green space or greenness within a defined jurisdiction of exposure [21]. In this study the first indicator of green space exposure was the percentage of land-use within each SA2 of residence covered by green space. Previous work has reported this indicator of green space quantity to be negatively associated with body mass index and positively associated with physical activity among children in the LSAC [22,23,24]. The green space land-use data was extracted from the Australian Bureau of Statistics “meshblock” classified as “parkland” in 2006. Meshblocks are the smallest set of geographical boundaries at which population data are available, constituting just 30 to 60 dwellings on average [18]. Meshblocks classified as “farmland” were not included in the definition of green space quantity as these are not typically areas for public access. Green space quantity in this study was expressed in categories of 0% to 20%, 20% to 40%, and >40%.

The second measure of green space exposure analysed in this study is green space quality. This indicator is atypical of current epidemiological literature on green space and health, aside from a small number of studies of adults [25,26,27,28]. Green space quality was reported by the spokes-parent through responding to the following statement: “*there are good parks, playgrounds and play spaces in this neighbourhood*”. Responses to the statement were either “strongly agreed”, “agreed”, “neither agreed nor disagreed”, “disagreed”, or “strongly disagreed”. Agreeable and disagreeable responses were aggregated to simplify the indicator, with the middle option (“neither agreed nor disagreed”) kept separate. 

### 2.4. Confounding Variables

The main source of confounding between child mental wellbeing and neighbourhood green space is socioeconomic circumstances, with children from disadvantaged families tending to have poorer wellbeing and living in areas where there is lower quantity or quality green space available [29,30]. To account for this and other potential sources of confounding, analyses were adjusted for indicators of area disadvantage and geographic remoteness, maternal education, child age and gender. Area disadvantage was measured using the Australian Bureau of Statistics “Socio Economic Index For Areas” (SEIFA) relative index of disadvantage. This indicator took into account variations between areas in a range of variables including unemployment, educational attainment and income [31]. The mother’s highest educational qualification was ascertained with responses including “postgraduate degree”, “graduate diploma or certificate”, “bachelor degree (including honours)”, “advanced diploma or diploma”, “certificate” or “other”.

### 2.5. Statistical Analysis

Descriptive statistics were used to examine the patterning of all of the variables across each of the green space quantity indicators. The outcome variables were skewed and exhibited over-dispersion, necessitating analysis via negative binomial regression. Two-level multilevel models with participants at level 1 and SA2s at level 2 were fitted for each of the nine outcome variables (three different indicators reported by three different informants). Models were initially fitted containing green space quantity, then green space quality, then a combination of both green space exposures followed by adjustment for confounders. Assessment of associations between each indicator of mental wellbeing in relation to green space quantity and quality were compared with respect to the three different informants in terms of the strength, direction and statistical significance. All models were fitted in MLwIN V2.30 (Centre for Multilevel Modelling, University of Bristol, Bristol, UK) [32] with parameters expressed as rate ratios (RR) with 95% confidence intervals (95% CI). Rate ratios above 1 denoted a positive association whereas below 1 indicated a negative association between outcome and exposure variables.

## 3. Results

Mean TDS scores tended to be higher when reported by children as compared to their parents (Table 1). In contrast, mean TDS scores reported by teachers or carers appeared to be substantially lower than those reported by children or parents. Mean TDS scores appeared to be lower among participants exposed to higher green space quantities regardless of the informant. Similar patterns were evident for the internalising and externalising subscales. Pearson correlation coefficients for TDS scores reported by parent and child were 0.4722 (*p* < 0.0001), parent and teacher 0.4907 (*p* < 0.0001), and child and teacher 0.4038 (*p* < 0.0001). Moderate levels of correlation between informants were also observed for the internalising and externalising subscales. Child age, gender and maternal education distributions did not vary substantially across strata of green space quantity. Participants living in remote and socially disadvantaged areas were more likely to be in the lowest strata of green space quantity. Agreement that local green spaces were of high quality was most common in areas with 20% to 40% green space quantity.

After adjustment for confounders, higher green space quantity was associated with lower TDS scores (Table 2), but the only statistically significant parameter was for >40% compared to 0% to 20% green space for TDS scores reported by parents (RR 0.890, 95% CI 0.815 to 0.972). Lower mean TDS scores were also lower for participants living near good quality green spaces regardless of the informant, though significantly only for parent-reported (RR 0.814, 95% CI 0.747 to 0.887) and child-reported TDS (RR 0.871, 95% CI 0.809 to 0.938). A similar pattern of results was observed for the internalising subscale (Table 3), with associations all in the anticipated direction, but the only statistically significant parameters were for parents (both green space quantity and quality) and children (quality only). For the externalising subscale (Table 4), higher green space quantity was associated with lower scores, but all parameters were not statistically significant. Very similar associations by parent report (RR 0.888, 95% CI 0.808 to 0.977) and child report (RR 0.888, 95% CI 0.821 to 0.961) were observed for green space quality and the externalising subscale, whereas no significant parameter was observed for the teacher-reported outcome.

## 4. Discussion

Prior studies of neighbourhood green space and child mental wellbeing, not unlike the wider research area of green space and health, have tended to rely on outcomes reported by single informants. For example, in Germany, Markevych and colleagues found poorer access to urban green spaces was associated with more behavioural problems—and hyperactivity/inattention problems in particular—among 10-year-old children whose parents had filled in the SDQ [11]. Balseviciene and colleagues’ study in Lithuania required mothers to fill in the SDQ on behalf of their four- to seven-year-old children, finding poorer mental wellbeing associated with lower residential greenness among those whose mothers had low levels of education [12]. Amoly and colleagues’ study of children aged seven to 10 years in Barcelona found fewer parent-reported SDQ-derived total difficulties, emotional symptoms and peer relationship problems among those spending more playing time within green spaces [13]. In England, Flouri and colleagues’ study observed fewer emotional problems as reported by parents on the SDQ for children aged three to five years old with access to more residential green space living in poorer communities, compared with their peers in more affluent areas [14]. The key finding from this study suggests that who the informant of the indicator of child mental wellbeing is can influence the results of an epidemiological study, despite the sample population and other characteristics being identical. The findings also suggest that weaker and non-significant results may be found in studies focusing upon child and/or teacher-reported SDQ-derived outcomes, instead of those reported by parents.

In general, the findings from this study support the theory that greener neighbourhoods and those where green spaces are considered to be of greater quality are promoters of child mental wellbeing. Stronger effect sizes were obtained from parent-reported outcomes in comparison to child and teacher informants. Key limitations include a cross-sectional design and potential confounding by factors at the person level (e.g., ethnicity) and the contextual level (e.g., excessive noise from heavy traffic).

Though analyses of child-reported outcomes tended to be in the anticipated direction, statistically significant findings were more consistently found in relation to green space quality than quantity. This is despite green space quality being a parent-reported variable and inherently subjective; the objective characteristics that determine whether a green space is high or low quality are likely to vary from parent to parent. The question used in this study is therefore advantageous in that it leaves the criteria for an assessment of quality open for the parent to interpret for themselves. However, there are limitations. First, the statement specifies parks, play spaces and playgrounds, the latter two of which are often found in parks, though not exclusively. There may be some cases where a parent refers to a good quality playground nearby in response to the aforementioned statement, which may not contain green space. While it is not known to what degree this is a problem, it is of course plausible that for some parents at least, the quality of a playground nearby may be diminished by a lack of greenery (and vice versa). Subjective reports of green space quality and potential associations with child health status warrant further investigation, but not just from parents as informants. The second limitation is that data were not available on whether the children themselves regarded local green spaces as good quality or not. This would be an interesting avenue to pursue in the future to contrast with parent-reported measures, especially since as children grow older, they tend to have greater autonomy and their spatial mobility without a parental chaperone increases markedly [33].

Although the findings from teachers were often in the anticipated direction but not statistically significant, this is relatively unsurprising given the fairly limited window of opportunity that they may have from which to draw their observations. This suggests that teacher-reported indicators of child mental wellbeing may be sub-optimal in studies of neighbourhood green space wherein parent- and or child-reported outcome data might be more preferable. That does not rule out the potential use-value of teacher-reported outcomes, however, if the study focus is on the level of greenery available specifically within the school context. Indeed, teacher-reported SDQs would appear to be a highly valuable outcome variable for studies that might involve some form of school-based intervention to increase exposure to green space. Some observational studies such as that of spectacle use among children have made attempts to capture exposure to green space within both the residential and school context [34], though studies that incorporate these cross-classifications with outcome variable data sourced from multiple informants remain rare. This is perhaps due to data availability and the relative complexity of modelling non-hierarchical data structures. 

Evidently, not all outcome variables have the potential to be reported by multiple informants, such as objectively measured body mass index or blood tests which are administered by trained clinical research assistants. Therefore, the findings from this study are applicable in many contexts but not all. The types of studies in which the findings from this analysis would be most relevant are the many examples of subjective outcome data among children (including but not limited to mental wellbeing) that could have multiple informants. Responses to whether or not a child participates in and/or enjoys physical activity, the number of friends they have, how long they sleep or watch television and how often they visit green spaces for recreational purposes is no exhaustive list, but all these factors could vary with respect to who is answering the question. Given that physical recreation and social contacts are considered to be important hypothesised pathways by which green space may support healthier lives, in addition to the restoration hypothesis which is directly linked to mental wellbeing, this is an important aspect of research that warrants more attention. Where outcome variables are reported by multiple informants, as can often be the case in studies of children, investigators are henceforth encouraged to replicate their analyses across each of those informants in order to explicitly test what difference they may make (if any) to the overall conclusions.

## 5. Conclusions 

The overall findings from this study support prior work that reports more favorable levels of mental wellbeing among children living within greener communities and/or near green spaces judged by their parents to be of good quality. However, another important finding was that the strength and statistical significance of those associations depended upon who was reporting the data used to construct the outcome variables. The most consistent associations were observed for parent-reported mental wellbeing. Few associations were observed for child-reported outcomes and none for those reported by teachers. This underlines the importance of studies in the area of green space and child health to acknowledge that different informants of outcome variables could lead to different results, conclusions and recommendations for policy-makers and practitioners.

## Figures and Tables

**Table 1:** Description of the study sample.

Green Space Quantity	0% to 20%	20% to 40%	>40%
*N*	1930	793	360
	**Mean (standard deviation)**		
Child mental wellbeing			
Total Difficulties Score			
Spokes-parent	7.42 (5.51)	7.07 (5.25)	6.47 (4.76)
Child	9.02 (5.52)	8.83 (5.47)	8.59 (5.69)
Teacher	5.88 (5.81)	5.58 (5.22)	5.52 (5.50)
Internalising subscale			
Spokes-parent	3.37 (3.02)	3.23 (3.03)	2.87 (2.68)
Child	3.88 (3.10)	3.73 (2.95)	3.62 (3.18)
Teacher	2.58 (2.99)	2.39 (2.67)	2.37 (2.80)
Externalising subscale			
Spokes-parent	4.05 (3.35)	3.84 (3.15)	3.60 (3.02)
Child	5.15 (3.36)	5.10 (3.38)	4.97 (3.46)
Teacher	3.30 (3.89)	3.19 (3.63)	3.15 (3.85)
Child age (years)	12.93 (0.31)	12.91 (0.31)	12.92 (0.31)
	**Frequency (percentage)**		
Child gender			
Male	972 (62.43%)	402 (25.82%)	183 (11.75%)
Female	958 (62.78%)	391 (25.62%)	177 (11.60%)
Maternal education			
Postgraduate degree	181 (64.64%)	70 (25.00%)	29 (10.36%)
Graduate diploma or certificate	160 (58.82%)	79 (29.04%)	33 (12.13%)
Bachelor degree	311 (59.46%)	146 (27.92%)	66 (12.62%)
Diploma or advanced diploma	222 (64.16%)	76 (21.97%)	48 (13.87%)
Certificate	555 (61.94%)	230 (25.67%)	111 (12.39%)
Other	28 (71.79%)	10 (25.64%)	1 (2.56%)
No response	473 (65.06%)	182 (25.03%)	72 (9.90%)
Geographic Remoteness			
Highly accessible	1181 (61.19%)	564 (29.22%)	185 (9.59%)
Accessible to moderately accessible	702 (63.99%)	227 (20.69%)	168 (15.31%)
Remote to very remote	47 (83.93%)	2 (3.57%)	7 (12.50%)
Area disadvantage			
Low (quintile 1)	584 (55.78%)	339 (32.38%)	124 (11.84%)
Moderate (quintile 2)	653 (62.55%)	276 (26.44%)	115 (11.02%)
High (quintile 3)	693 (69.86%)	178 (17.94%)	121 (12.20%)
Local green spaces good quality			
Disagree or strongly disagree	209 (69.67%)	56 (18.67%)	35 (11.67%)
Agree or strongly agree	1495 (61.47%)	652 (26.81%)	285 (11.72%)
Ambivalent or no response	226 (64.39%)	85 (24.22%)	40 (11.40%)

**Table 2:** Associations between indicators of objectively measured green space quantity, parent-reported green space quality, and Strengths and Difficulties Questionnaire Total Difficulties Scores for children aged 12–13 years as reported by their spokes-parent, themselves and their teacher.

Variable	Model 1 (Spokes-Parent)	Model 2 (Child)	Model 3 (Teacher)
	Rate Ratio (95% Confidence Interval)		
Child age (years)	1.043 (0.961, 1.131)	**1.099 (1.025, 1.177)**	**1.167 (1.049, 1.298)**
Gender (ref: Male)			
Female	**0.780 (0.742, 0.820)**	0.967 (0.927, 1.010)	**0.597 (0.559, 0.638)**
Maternal education (ref: postgrad)			
Graduate diploma or certificate	0.935 (0.829, 1.055)	0.996 (0.898, 1.104)	0.971 (0.829, 1.138)
Bachelor degree	**0.895 (0.806, 0.994)**	0.940 (0.859, 1.029)	0.897 (0.781, 1.030)
Diploma or advanced diploma	1.005 (0.897, 1.125)	1.027 (0.931, 1.131)	0.995 (0.857, 1.156)
certificate	**1.144 (1.037, 1.261)**	**1.147 (1.054, 1.247)**	**1.181 (1.038, 1.343)**
other	0.837 (0.654, 1.071)	1.163 (0.949, 1.424)	1.075 (0.785, 1.473)
Geographic remoteness (ref: highly accessible)			
Moderately accessible	**0.936 (0.879, 0.996)**	0.965 (0.918, 1.014)	0.934 (0.863, 1.010)
Remote	1.025 (0.829, 1.268)	1.070 (0.911, 1.257)	1.195 (0.925, 1.545)
Area disadvantage (ref: low)			
Moderate	**1.096 (1.021, 1.177)**	**1.097 (1.037, 1.160)**	**1.118 (1.023, 1.221)**
High	**1.163 (1.078, 1.255)**	**1.145 (1.077, 1.216)**	**1.232 (1.120, 1.355)**
Green space quantity (0% to 20%)			
20% to 40%	0.978 (0.917, 1.044)	1.008 (0.958, 1.061)	0.986 (0.910, 1.070)
>40%	**0.890 (0.815, 0.972)**	0.971 (0.906, 1.041)	0.964 (0.864, 1.075)
Green space good quality (ref: Disagree or strongly disagree)			
Agree or strongly agree	**0.814 (0.747, 0.887)**	**0.871 (0.809, 0.938)**	0.906 (0.808, 1.016)

**bold** *p* < 0.05.

**Table 3:** Associations between indicators of objectively measured green space quantity, parent-reported green space quality, and Strengths and Difficulties Questionnaire internalising subscale for children aged 12–13 years as reported by their spokes-parent, themselves and their teacher.

Variable	Model 4 (Spokes-Parent)	Model 5 (Child)	Model 6 (Teacher)
	Rate Ratio (95% Confidence Interval)		
Child age (years)	0.982 (0.887, 1.088)	1.040 (0.949, 1.138)	1.073 (0.942, 1.221)
Gender (ref: Male)			
Female	**0.920 (0.864, 0.980)**	1.106 (1.046, 1.170)	**0.804 (0.743, 0.872)**
Maternal education (ref: postgrad)			
Graduate diploma or certificate	0.963 (0.828, 1.121)	0.938 (0.819, 1.075)	0.958 (0.789, 1.161)
Bachelor degree	0.893 (0.783, 1.019)	0.905 (0.805, 1.018)	0.925 (0.782, 1.093)
Diploma or advanced diploma	0.953 (0.827, 1.099)	0.973 (0.856, 1.105)	0.987 (0.823, 1.184)
certificate	1.093 (0.967, 1.236)	1.105 (0.991, 1.233)	1.128 (0.965, 1.319)
other	0.732 (0.531, 1.009)	1.069 (0.819, 1.395)	1.023 (0.697, 1.501)
Geographic remoteness (ref: highly accessible)			
Moderately accessible	0.932 (0.864, 1.007)	0.967 (0.906, 1.032)	0.942 (0.856, 1.036)
Remote	1.104 (0.858, 1.420)	1.059 (0.859, 1.305)	1.180 (0.864, 1.610)
Area disadvantage (ref: low)			
Moderate	**1.118 (1.025, 1.219)**	**1.148 (1.067, 1.235)**	**1.170 (1.050, 1.304)**
High	**1.142 (1.041, 1.254)**	**1.167 (1.078, 1.264)**	**1.243 (1.107, 1.397)**
Green space quantity (0% to 20%)			
20% to 40%	0.987 (0.912, 1.068)	0.992 (0.927, 1.060)	0.968 (0.877, 1.069)
>40%	**0.867 (0.778, 0.967)**	0.948 (0.865, 1.038)	0.942 (0.824, 1.077)
Green space good quality (ref: Disagree or strongly disagree)			
Agree or strongly agree	**0.740 (0.665, 0.822)**	**0.855 (0.777, 0.940)**	0.877 (0.764, 1.007)

**bold** *p* < 0.05.

**Table 4:** Associations between indicators of objectively measured green space quantity, parent-reported green space quality, and Strengths and Difficulties Questionnaire externalising subscale for children aged 12–13 years as reported by their spokes-parent, themselves and their teacher.

Variable	Model 7 (Spokes-Parent)	Model 8 (Child)	Model 9 (Teacher)
	Rate Ratio (95% Confidence Interval)		
Child age (years)	1.093 (0.998, 1.196)	**1.144 (1.062, 1.231)**	**1.252 (1.099, 1.426)**
Gender (ref: Male)			
Female	**0.681 (0.644, 0.720)**	**0.875 (0.836, 0.916)**	**0.465 (0.428, 0.504)**
Maternal education (ref: postgrad)			
Graduate diploma or certificate	0.911 (0.795, 1.044)	1.038 (0.929, 1.160)	0.962 (0.791, 1.169)
Bachelor degree	0.900 (0.800, 1.013)	0.968 (0.878, 1.066)	0.857 (0.722, 1.016)
Diploma or advanced diploma	1.062 (0.936, 1.205)	1.071 (0.964, 1.188)	1.003 (0.834, 1.205)
certificate	**1.197 (1.073, 1.335)**	**1.175 (1.074, 1.286)**	**1.225 (1.045, 1.435)**
other	0.930 (0.708, 1.223)	1.220 (0.984, 1.512)	1.132 (0.770, 1.665)
Geographic remoteness (ref: highly accessible)			
Moderately accessible	**0.950 (0.886, 1.018)**	0.964 (0.913, 1.018)	0.927 (0.843, 1.018)
Remote	0.969 (0.765, 1.228)	1.082 (0.905, 1.294)	1.206 (0.893, 1.627)
Area disadvantage (ref: low)			
Moderate	**1.083 (1.001, 1.172)**	1.062 (0.998, 1.130)	1.069 (0.961, 1.189)
High	**1.187 (1.091, 1.291)**	**1.130 (1.057, 1.207)**	**1.220 (1.089, 1.368)**
Green space quantity (0% to 20%)			
20% to 40%	0.971 (0.903, 1.044)	1.021 (0.965, 1.080)	0.993 (0.902, 1.094)
>40%	0.907 (0.822, 1.000)	0.984 (0.912, 1.062)	0.963 (0.845, 1.098)
Green space good quality (ref: Disagree or strongly disagree)			
Agree or strongly agree	**0.888 (0.808, 0.977)**	**0.888 (0.821, 0.961)**	0.934 (0.811, 1.074)

**bold** *p* < 0.05.
